# A Study on Machining Performances of Micro-Drilling of Multi-Directional Carbon Fiber Reinforced Plastic (MD-CFRP) Based on Nano-Solid Dry Lubrication Using Graphene NanoPlatelets

**DOI:** 10.3390/ma14030685

**Published:** 2021-02-02

**Authors:** Jin Woo Kim, Jungsoo Nam, Jaehun Jeon, Sang Won Lee

**Affiliations:** 1 Department of Mechanical Engineering, Graduate School, Sungkyunkwan University, Suwon-si 16419, Korea; jw9008@skku.edu (J.W.K.); hoon3159@skku.edu (J.J.); 2 Intelligent Manufacturing System R&D Department, Korea Institute of Industrial Technology, Cheonan-si 31056, Korea; rack1219@kitech.re.kr; 3 School of Mechanical Engineering, Sungkyunkwan University, Suwon-si 16419, Korea

**Keywords:** micro-drilling, multi-directional carbon fiber-reinforced plastic (MD-CFRP), nano-solid dry lubrication, tribological behavior, machining performance, graphene nanoplatelets

## Abstract

The objective of this study is to investigate the tribological behavior of graphene nanoplatelets (xGnPs) as nano-solid lubricants, and to evaluate their applicability to the micro-drilling of multi-directional carbon fiber-reinforced plastic (MD-CFRP). To verify the tribological effect of nano-solid lubricants, three kinds of xGnPs (xGnP C-750, xGnP M-5, and xGnP H-5), multiwall carbon nanotubes (MWCNTs), and hBN are compared by the ball-on-plate test. Of these, three xGnPs are selected as nano-solid lubricants to investigate the micro-drilling performance of MD-CFRP using nano-solid dry lubrication, and the experimental results demonstrate that all xGnPs can enhance lubrication action in terms of surface quality (delamination, uncut fiber, and inner surface) and tool wear. In particular, larger graphene nanoplatelets (xGnP M-5 and xGnP H-5) are superior to the smaller one (xGnP C-750) by guaranteeing enhanced sliding action between the tool grain and the CFRP composite.

## 1. Introduction

Generally, a carbon fiber-reinforced plastic (CFRP) composite that can be fabricated by combining carbon fiber and polymer matrix has been used for high-tech applications, such as in the automobile and aerospace industries. The CFRP composite is generally known as a superior metal substitute due to its advanced properties, such as superior strength-to-weight ratio, stiffness, damage tolerance, and so on [1,2].

However, because of its complicated structure, which is represented by anisotropy and non-homogeneity, the use of CFRP in machining processes can lead to severe surface defects such as delamination, fiber pull-out [3,4]. In addition, due to frictional force and high temperature at the tool-work interface, excessive tool wear occurs [5]. Therefore, to enable efficient machining with CFRP composite, there is a demand for an appropriate lubrication approach.

When machining metallic materials, metal working fluids (MWFs) has typically been used as the types of wet flooding and minimum quantity lubrication (MQL) for reducing high cutting heat, and improving lubrication performance, respectively. To further enhance machining performance, nano-scale particles have been used as nanofluids by adding them to MWFs and liquid medium to help the lubrication action during the machining process, and their effectiveness in nanofluid MQL machining on metallic materials has been verified [6,7,8].

However, it has been reported that CFRP composite has moisture-sensitive properties, and long-term exposure to liquid could negatively affect its excellent mechanical properties [9,10,11]. Moreover, there is a lack of research on appropriate lubrication technologies that could prevent degradation of CFRP’s mechanical properties, and it was also found that the wet machining might worsen tool life and hole quality during the CFRP drilling [12,13].

Considering the moisture-sensitive nature of CFRP, the author’s research group has recently proposed a novel lubrication technique that has been named the nano-solid dry lubrication technique [14]. With this new method, air-driven nano-scale particles can be sprayed into the cutting zone. In this study, one kind of graphene nanoplatelets (xGnP H-5) and multiwall carbon nanotubes (MWCNTs) were used as nano-solid lubricants, respectively, and their machining performances on uni-directional CFRP (UD-CFRP) were analyzed in terms of surface quality and tool wear. The results showed that xGnP H-5 was more advantageous than MWCNT.

In a previous study, an analysis considering the tribological behavior of nano-solid lubricants was not conducted. To elucidate the lubrication mechanism of carbon allotrope particles, it is necessary to analyze the tribological properties of the nanoparticles. In various studies involving tribological tests, the types of using carbonous lubricants used to lubricate the two-body interface can be classified into four groups (I. specimen coating, II. specimen reinforcement as additive, III. nanofluid, and IV. solid powder). <I. specimen coating>: Le et al. used multiwall carbon nanotubes for surface coating in the reciprocating sliding test, and this coating could help achieve anti-corrosion and low friction [15]. <II. specimen reinforcement as additive>: Moghadam et al. (2015) reviewed the mechanical and tribological properties of a material reinforced by carbon nanotubes and graphene. The results confirmed that adding carbon nanotubes and graphene to metal reduced both the coefficient of friction and the wear rate due to self-lubricating properties of these carbonous particles [16]. <III. nanofluid>: Berman et al. conducted the ball on disk test using graphene-containing ethanol solution, and this resulted in low wear, easy shearing, and reduced friction by acting as a protective coating [17]. <IV. solid powder>: Yin et al. suggested that sprinkling solid graphene lubricant on the steel wafer surface can effectively reduce the coefficient of friction and wear by forming carbon-based tribofilm during the ball on plate test [18].

As mentioned in those studies, these carbonous particles were verified to lead to effective lubrication, but no results were found in terms of the lubricity for CFRP as nano-solid lubricants. Therefore, the objective of this study is to investigate direct tribological behavior of nano-solid lubricants against multi-directional carbon fiber-reinforced plastic (MD-CFRP), and to evaluate their applicability to the micro-drilling of MD-CFRP considering three different sizes of xGnPs.

In this paper, to analyze tribological behavior, nano-solid lubricants are sprinkled on the specimen as in the <IV. solid powder> considering the moisture-sensitiveness of CFRP composite, and the tribological characteristics are investigated in terms of the coefficient of friction (COF) and wear. In this test, using the ball on plate equipment, three different sizes of xGnPs (xGnP C-750, xGnP M-5, and xGnP H-5) are analyzed, and MWCNTs and hexagonal boron nitride (hBN) are also tested to compare performance.

Finally, in the micro-drilling of MD-CFRP using nano-solid dry lubrication, the three xGnPs are used to evaluate machining performance. In total, there are five experimental cases, including the test cases of dry and pure air lubrication. The experimental results are evaluated in terms of delamination, uncut fiber, inner surface quality, and tool wear. The lubrication mechanism is analyzed by reflecting the actual sizes of the graphene nanoplatelets and the tool grain. As a result, it is concluded that the larger graphene nanoplatelets (xGnP M-5 and xGnP H-5) are more effective for lubricating action between tool-workpiece interfaces by guaranteeing a sufficient sliding effect.

## 2. Tribological Test of Nano-Solid Lubricants

Prior to the micro-drilling experiments, to confirm the effects of the graphene nanoplatelets as lubricants against CFRP composites, a series of triobological tests was conducted via the ball on plate test, and the coefficient of friction and magnitude of wear were analyzed. In addition to the graphene nanoplatelets, multiwall carbon nanotubes (MWCNTs) and hexagonal boron nitride (hBN) were also investigated for a comparison. As a result, five nano-solid lubricants were used in total for the tribological test.

### 2.1. Properties of Nano-Solid Lubricants

**Figure 1:**
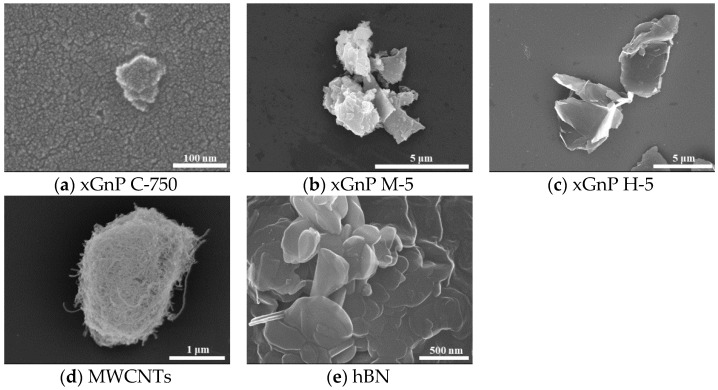
FE-SEM images of xGnPs, MWCNTs, and hBN.

The structures and shapes of the nanoparticles were confirmed by using field emission scanning electron microscope (JSM-7600F, JEOL Ltd., Tokyo, Japan), and the images are shown in Figure 1. In this paper, while taking field emission scanning electron microscope (FE-SEM) images, the acceleration voltage was 15.0 kV. In addition, the platinum (Pt) sputtering was conducted for minimizing a charging effect.

The properties of the five nano-solid lubricants are summarized in Table 1. The three different graphene nanoplatelets called xGnP manufactured by XG SCIENCE were classified as xGnP C-750, xGnP M-5, and xGnP H-5. The xGnP C-750 was categorized as C grade with an average particle size (APS) of ~0.2 μm and a specific surface area (SSA) of 750 m^2^/g. The xGnP M-5 was larger than the xGnP C-750 and had an APS of 5 μm as well as a thickness of 8 nm. The xGnP H-5 was the largest of the three graphene nanoplatelets, and it had an APS of 5 μm and a thickness of 15 nm. It was confirmed that the three xGnP particles had a form of stacks of thin two-dimensional (2D) sheets in common.

In addition, MWCNTs (US4500) manufactured by US Research Nanomaterials, Inc. (Houston, TX, USA) which consist of carbon (C) allotropes such as xGnPs but with a tube-shape structure were confirmed, and the hBN (MK-hBN-070) from M K Impex Corp. (Mississauga, Canada), which is simply known as white graphene with an analogous structure to xGnP by bonding boron (B) and nitrogen (N), are presented and summarized in Figure 1 and Table 1.

**Figure 2:**
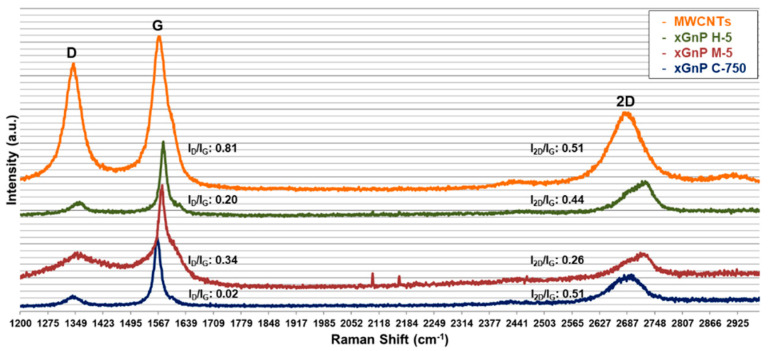
Raman spectra of xGnPs and MWCNTs.

In order to confirm the degree of crystallinity and the number of graphene layers, Raman spectroscopy equipment (Micro-Raman Spectroscopy System, RENISHAW, Wotton-under-Edge, England) was used. In Figure 2, Raman spectra of xGnPs and MWNCTs are given. Generally, the D band (1300–1400 cm^−1^) is related to the edge disorder and defects in carbon structure, and the G band (1550~1615 cm^−1^) is known as the graphitic region. The 2D band (~2700 cm^−1^) is an overtone of the D band, and its asymmetric shape is the index of multi-layer of graphene. The ratio between intensity of the D band and the G band (I_D_/I_G_) is used to an indicator of crystallinity of the graphene layer. The ratio between intensity of the 2D band and G band (I_2D_/I_G_) explains the number of graphene layers. In Figure 2, it was found that MWCTNs in the form of tube-shape had larger I_D_/I_G_ than those of xGnPs, indicating a decrease in the degree of crystallinity of the graphene layer. When comparing the I_2D_/I_G_ of xGnPs, the values less than 1 was confirmed, which means the multi-layer graphene (graphite). In addition, the asymmetry of the 2D bands was confirmed in the large xGnPs (xGnP M-5 and xGnP H-5) compared to smaller one (xGnP C-750) [22,23,24,25].

### 2.2. Tribological Test Design and Conditions

**Figure 3:**
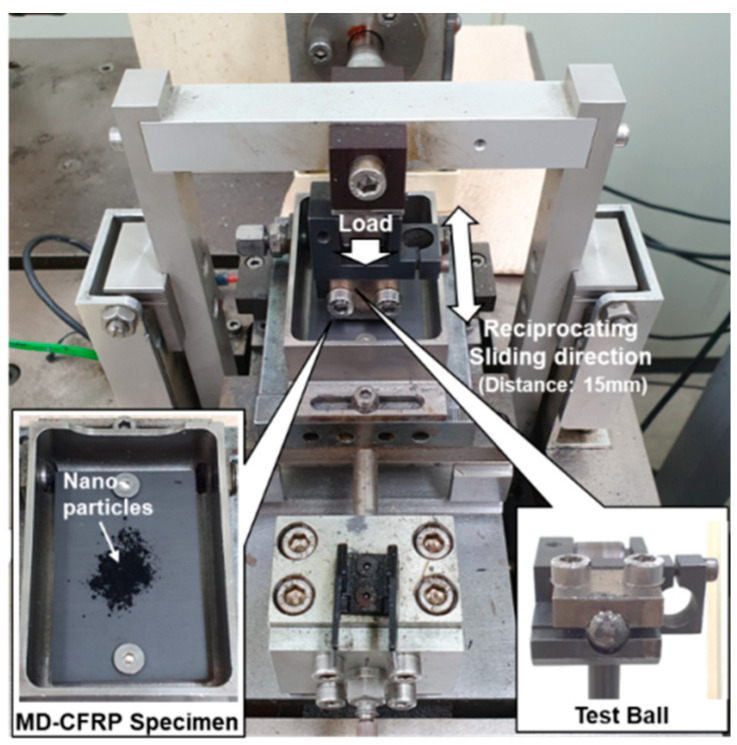
Ball on plate test equipment.

In order to investigate the tribological properties of nano-solid lubricants against MD-CFRP composites, reciprocating ball on plate equipment (TE77 AUTO, Phoenix Tribology Ltd., Kingsclere, England) was used, and the coefficient of friction (COF) data were measured in each test case. Figure 3 shows the ball on plate equipment containing the test specimen and the test ball.

The test specimen was fabricated with MD-CFRP, which is prepared by laminating prepreg with a carbon fiber of T700 (Toray Composite Materials America, Inc., Tacoma, WA, USA) with 13-ply, and the size was 38 mm × 58 mm × 4 mm. The stacking sequence of the MD-CFRP specimen was [0°/0°/90°/90°/90°/0°/0°/0°/90°/90°/90°/0°/0°]_s_, and information on the carbon fiber and the prepreg is listed in Table 2. The test ball was tool steel with a diameter of 10 mm, and a new test specimen and ball were used for each experiment.

In total, six experimental cases were tested, consisting of a dry case without lubricants, xGnP C-750, xGnP M-5, xGnP H-5, MWCNTs, and hBN. Except for the dry case, 0.05 g of lubricants was sprinkled on each MD-CFRP specimen. The load of the test ball (normal force) was 50 N, and the sliding distance was 15 mm. In addition, the reciprocating speed and the total test time were 2 Hz, and 610 s, respectively.

### 2.3. Tribological Test Results

**Figure 4:**
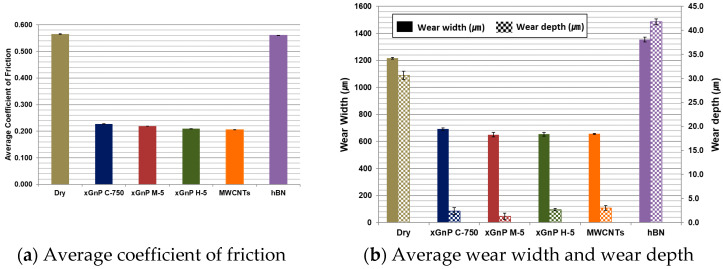
Average coefficient of friction and wear values in each case.

In order to analyze the tribological behaviors of nano-solid lubricants on the CFRP composite, the coefficient of friction (COF) and the wear on the specimen were investigated. In each case, the COF value was averaged for 180 sec after the test was stabilized. The average COF values according to different nano-solid lubricants are given in Figure 4a, and these are compared with that of the dry case. As shown in the graph, the highest value was obtained in the dry case without lubricants. In the cases of the three xGnPs and the MWCNTs, the values were significantly reduced. On the other hand, the COF in the case of hBN showed little difference compared to the dry case. In Figure 4b, the average wear width and depth values are given. Similar to the COF results, the wear width and depth of the specimen was substantially smaller in the cases of xGnPs and MWCNTs composed of graphene-based particles. On the other hand, they were both highest in the case of hBN.

**Figure 5:**
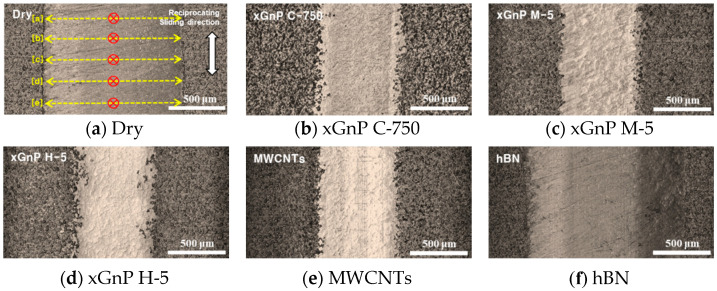
Optical microscopic images of MD-CFRP after the ball on plate test.

These wear values were measured by obtaining images of the specimen and 3D profile data by using a 3D laser microscope (VK-X series, Keyence, Osaka, Japan). The optical microscopic photos of each test case are shown in Figure 5. For more detailed analysis, 5 sectors ([a]–[e]) were selected, and they were 150 μm apart vertically from each other. The direction of measurement of wear width was expressed to yellow color, and the maximum wear depth was expressed to red color, as representatively shown in Figure 5a. In each case, the wear width and depth were measured and averaged using the VK Analyzer software.

**Figure 6:**
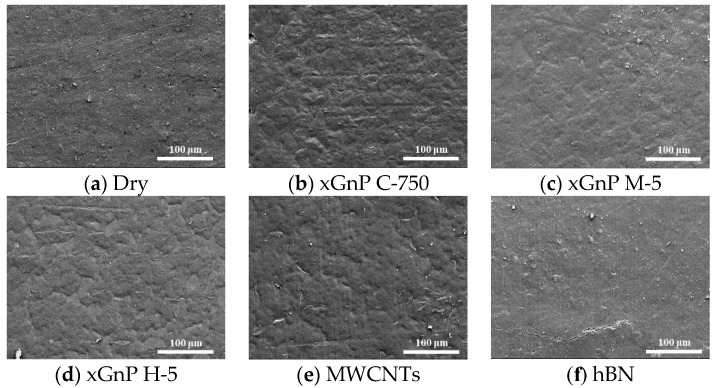
FE-SEM images of worn surfaces during the ball on plate test (×300).

For more detailed analysis, FE-SEM images of the worn surfaces were taken, and their morphological characteristics were studied. In Figure 6, the FE-SEM images for each case are shown, and the surface morphologies in the cases of three xGnPs and MWCNTs were quite similar one another. It seems that the entire surfaces were covered with continuous film. Therefore, it is assumed that the lubrication mechanism of these graphene-based particles could be similar. On the other hand, in the cases of dry and hBN, such continuous film is not observed, and the worn surfaces are rougher and has cracks. In Section 2.4, further scientific explanations are given.

### 2.4. Discussions

**Figure 7:**
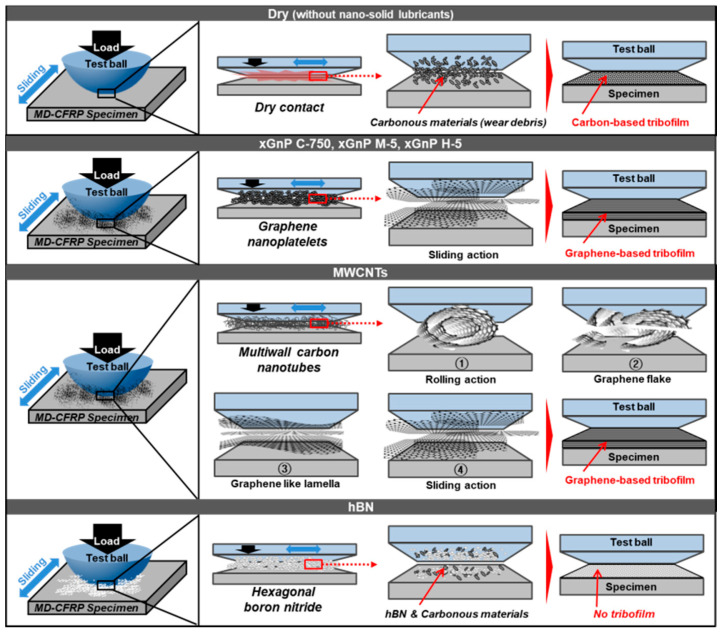
Primary lubrication mechanism in each case during the ball on plate test.

In these tribological test results, the three graphene nanoplatelets (xGnP C-750, xGnP M-5, and xGnP H-5) and the multiwall carbon nanotubes (MWCNTs) could meaningfully reduce the coefficient of friction and the wear width and depth. However, hexagonal boron nitride (hBN) was not effective. To elucidate the primary lubrication mechanism for each case reasonably, the FE-SEM images given in Figure 6 were more deeply investigated by referencing some studies on tribological characteristics of carbonous materials. The analyzed schematic expressions are provided in Figure 7.

It has been known that carbonous materials basically have the self-lubricating property. Suresha et al. reported a self-lubricating nature of carbon fiber from the abrasion test [27]. Zhu et al. conducted the pin joint test of graphite and carbon fiber modified polymer, and the self-lubricating carbonous characteristics were found by observing worn surfaces and wear debris [28]. In addition, these carbonous materials could create tribofilm. Zhao et al. found that short carbon fibers improve the anti-wear properties of polymer composites during the block-on-ring test by forming tribofilm [29]. Luo et al. found the formation of transfer film of polymer-carbon fiber composite by acting dry-sliding during the block-on-ring test [30].

Therefore, in the dry case, wear debris occurred by the breakage of the carbon fiber which composes the MD-CFRP specimen could stay between the two counterparts. These carbonous materials could have formed carbon-based tribofilm due to the repeated stressing cycles. As a result, dry-sliding behavior occurred due to the self-lubricating capability of carbonous materials in MD-CFRP, despite the lack of nano-solid lubricants.

In this context, the carbonous nano-solid lubricants could enhance such lubricating capability even more. In the cases of the three xGnPs, it was analyzed that the very thin two-dimensional graphene layers (general thickness of single-layer graphene: ~0.34 nm) slid on each other by enabling proper lubrication action. The lubrication characteristics of xGnP C-750, xGnP M-5, and xGnP H-5 were difficult to distinguish from each other, as shown in the test results. This could have been because the particles were rubbed by the continuous movement of reciprocating sliding while loading the normal force on the test ball, so analogous graphene-based tribofilm could be formed in all xGnP cases by increasing the contact pressure between the test ball and the specimen.

In the case of MWCNTs, the tube-shape structure might have been maintained at the beginning of the lubrication phase, despite the load on the test ball and the sliding motion. However, it was analyzed that the multiwall tube-shape structure consisting of graphene was deformed from outside the structure through contact pressure, and graphene flakes started to fall off, resulting in breakage of the covalent bonds. The damaged MWCNTs were degraded to a two-dimensional hexagonal graphene structure such as xGnPs, and it is assumed that a kind of graphene-based tribofilm was ultimately formed. The evidence for the deformation and lubrication mechanism of MWCNTs was found from several tribological studies. Reinert et al. proposed a structural degradation model of the mechanically stressed MWCNTs by using the custom-made ring-on-block tribometer [31]. Sakka et al. investigated the friction and wear of bulk epoxy and carbon filler reinforced epoxy composites using the pin-on-disc tribometer and presented the schematic diagram of structural transformation of MWCNTs [32].

As discussed in the previous section, the surface morphologies that seem to be added layers are observed in Figure 6b–e, and they could play roles of tribofilm during the ball on plate test. Therefore, the proposed lubrication mechanism given in Figure 7 could be confirmed.

Meanwhile, as indicated by the test results, the hBN case had worse wear values than the dry case. In this case, there existed ceramic materials, hBN, and carbonous debris created by the friction test. As the two materials mixed with each other, the lubricity was analyzed to be lower than that of the dry case by making three-body contact instead of creating tribofilm. Therefore, it was confirmed that there was no lubrication effect as the solid lubricants, resulting in substantially more wear than in the dry case.

As shown by the test results on friction and wear, the cases of the three xGnPs and the MWNCTs could improve the lubrication behavior compared to the dry case while forming graphene-based tribofilm by reducing the direct contact between the ball and the CFRP specimen. Moreover, in these cases, the effect of the self-lubricating property was maximized due to the graphene-based nanoparticles.

## 3. Micro-Drilling Experiments with Nano-Solid Lubrication

To benefit from the enhanced tribological effect of carbonous nano-scale particles, three xGnP particles were chosen for the nano-solid dry lubrication of micro-drilling experiments. These particles had a shape of two-dimensional thin sheet, and it was expected that they could significantly improve lubrication in micro-drilling of a CFRP workpiece with their inherent sliding action.

### 3.1. Experimental Set-Up

In this research, a multi-directional CFRP (MD-CFRP) composite was used as the workpiece for micro-drilling process. The size of the workpiece was 30 mm × 30 mm × 3 mm. The MD-CFRP workpiece was fabricated with 11-ply in the directions of 0° and 90°. The stacking sequence of the MD-CFRP composite was [90°/0°/0°/0°/90°/90°/90°/0°/0°/0°/ 90°]_s_, and the carbon fiber of prepreg used in this workpiece was the same one used for the tribological test (Table 2 in Section 2.2).

**Figure 8:**
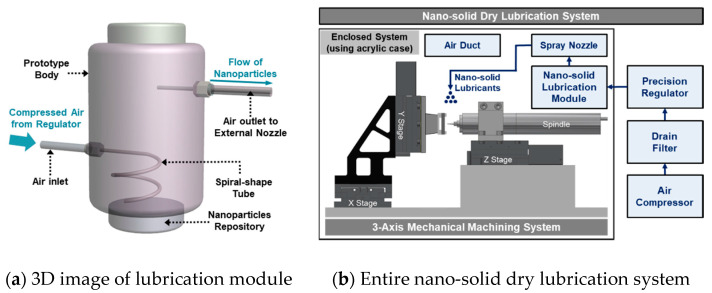
Nano-solid lubrication module and schematic diagram of entire nano-solid dry lubrication system.

To apply the nano-solid dry lubrication technique, a nano-solid lubrication spray module was designed and is shown in Figure 8a. The module has an air inlet and an air outlet. A spiral-shape tube was used in an attempt to decrease the aggregation of the nano-solid lubricants, considering the cohesion of particles. For lubrication action, the air-driven lubricants were sprayed to the cutting zone via an external nozzle. The customized lubrication module was integrated to construct the entire nano-solid lubrication system. An air compressor (PAB 42T3-503F, Power Air, Yangpyeong-gun, Korea), a drain filter (WD-02, Win & Tec Korea, Anyang-si, Korea), a precision regulator (IR 1000-01G, SMC Coporation, Tokyo, Japan), and an air duct were set to help spray the nano-solid dry lubricants, as shown in Figure 8b.

**Figure 9:**
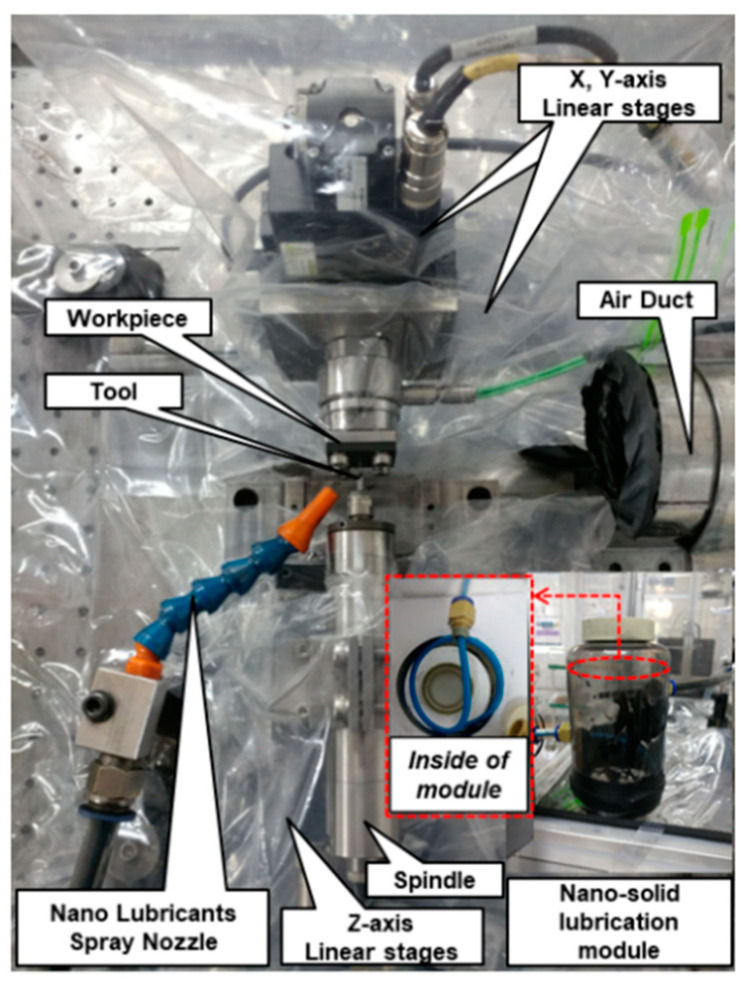
Experimental set-up for MD-CFRP machining using nano-solid dry lubrication.

The three-axis horizontal machine tool system with a nano-solid dry lubrication module is shown in Figure 9, and several components with three DC-motor driven linear slides (MX80S, Parker-Hannifin Corporation, Mayfield Heights, OH, USA), electric spindle (E-800Z, Nakanishi, Kanuma, Japan) and programmable multi-axis controller (CEM 104, Delta Tau Data Systems, Chatsworth, CA, USA) were prepared. An external spray nozzle with an internal diameter of 3 mm was set 17 mm away from the workpiece with a spraying elevation angle of 60° for nano-solid dry lubrication. A two-flute uncoated tungsten carbide drill (DIXI 1138, Dixi Polytool, Le Locle, Switzerland) with a diameter of 800 μm was mounted to the electric spindle.

### 3.2. Experimental Design and Conditions

In order to improve the micro-drilling performance of MD-CFRP, three graphene nanoplatelets (xGnP) were used as nano-lubricants: xGnP C-750, xGnP M-5, and xGnP H-5. The properties of xGnPs are summarized in Figure 1 and Table 1 in Section 2.1. In total, there were five experimental cases, including dry and pure air cases. In each case, a new tool and workpiece was used, and 92 instances of through-hole drilling were conducted with a hole depth of 3 mm. The drilling parameters, being the spindle speed, feed per revolution that were used in this paper were carefully chosen by considering the recommended drilling conditions of the manufacturer of the drill [33]. As a result, the spindle speed and feed per revolution were fixed at 20,000 RPM and 14 μm/rev, respectively, after several preliminary drilling experiments. In addition, the air pressure was 0.4 bar for the pure air and xGnPs nano-solid lubrication.

## 4. Micro-Drilling Performance Evaluation–Methods and Analysis

To evaluate machining performances of the micro-drilling of MD-CFRP based on nano-solid dry lubrication using three graphene nanoplatelets, the general machining performance factors of CFRP composite drilling were considered [34,35,36,37]. The experimental results were comprehensively discussed in terms of surface quality (delamination, uncut fiber and inner surface) and tool wear.

### 4.1. Delamination

To analyze the quality of outside of the drilled holes, the delamination at an exit hole was considered. The delamination can be occurred by the push-down mechanism. When the drilling thrust force exceeds the interlaminar bond strength before the CFRP laminate is totally drilled by the tool, this push-down can occur and result in the delamination. Thus, the delamination factor (F_d_) explains the degree of compressive thrust force that the drill bit can penetrate on the CFRP [38].

**Figure 10:**
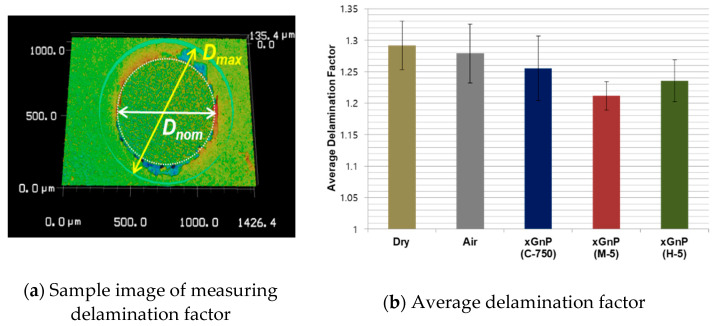
Sample images for calculating delamination and average delamination factor.

The factor can be calculated by taking the ratio of the maximum diameter, D_max_, with respect to the nominal diameter, D_nom_, of the micro-drilled hole. In the experiments, the maximum diameter, D_max_, is the maximum extension of delamination outside the drilled hole and the nominal diameter, D_nom_, is equal to the tool diameter of 800 μm. To analyze the delamination, a 3D laser microscope (VK-X series, Keyence, Osaka, Japan) and processing software were used. Figure 10a shows an image of a sample drilled hole with D_nom_ and D_max_ for calculating the delamination factor.

For the analysis of each experimental case, delamination factors were compared at the same drilled hole. The delamination factors for 12 holes–the 5th, 15th, 23rd, 28th, 38th, 46th, 51st, 61st, 69th, 74th, 84th, and 92nd holes out of 92 were calculated at the exit hole, and their average values are shown in Figure 10b.

As shown in Figure 10b, the average delamination factor in the dry case was substantially higher than those of the other cases. Meanwhile, pure air, xGnP C-750, xGnP M-5, and xGnP H-5 nano-solid lubrication could reduce the delamination of the exit hole. In particular, xGnP M-5 could be the best option for decreasing the delamination factor, while xGnP H-5 also seems to be effective for reducing the delamination factor. In addition, pure air also could reduce the delamination factor over that of the dry case. Therefore, it was confirmed that nano-solid lubrication using graphene nanoplatelets could significantly enhance the machining performance of MD-CFRP composite in terms of the reduction of the delamination factor.

### 4.2. Uncut Fiber

The mechanism of delamination and uncut fiber are interdependent. To investigate the quality of inside of the drilled holes, the uncut fiber area was studied at exit hole. The uncut fiber was generated more frequently in certain fiber cutting angles. It was known that the uncut fiber was generated at cutting angles between 90–180°, as the protrusion direction of the fibers parallel to the cutting edge, tool could cut the CFRP resin more easily than the fibers which has a higher tensile strength. In particular, at a cutting angle of 135°, the maximum uncut fiber usually occurs. On the other hand, at cutting angles between 0–90°, the cutting is appropriately done by the shear stress as the force is applied to the side of fiber when the tool rotates clockwise [39].

**Figure 11:**
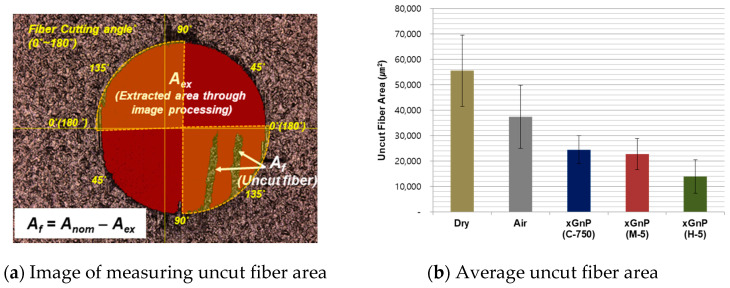
Sample images for measuring uncut fiber and average uncut fiber area.

Figure 11a shows the image of measuring the uncut fiber area, and the cutting angles between 90–180° were expressed in a yellow color, observing uncut fiber. The uncut fiber area was estimated in this study using the image processing software (VK Analyzer, Osaka, Japan). The uncut fiber area, A_f_, is the difference between the nominal drilled-hole area, A_nom_, and the extracted area through image processing, A_ex_; A_nom_ is the area of a circle with a diameter of 800 μm. A_ex_ for calculating the uncut fiber area was extracted as a red color through image processing. The processing parameter was set with the tolerance of the RGB data of 10.

The values of the calculated uncut fiber area for 12 holes were averaged, which was the same hole used for the delamination factor, and these are graphically provided in Figure 11b. As expected, the uncut fiber area value in the dry case was the highest when compared with those of other cases. Similar to the result of analyzing the delamination factor, it was found that nano-solid lubrication could reduce the uncut fiber area more than the sole air supply case. Meanwhile, the smallest value was confirmed in the case of xGnP H-5. This suggests that xGnP H-5 is more effective for reducing uncut fiber area than xGnP C-750.

**Figure 12:**
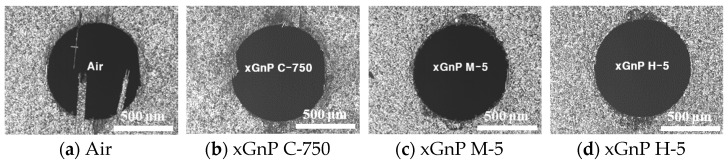
Images of the 28th drilled exit hole of MD-CFRP in each experimental case.

To qualitatively compare the quality of the drilled holes, optical images of the 28th hole were taken in the case of four different types of lubrication, and these are shown in Figure 12. In the case of pure air without graphene nanoplatelets, remarkable delamination and uncut fiber defects were observed at the drilled hole. On the other hand, with xGnP M-5 and xGnP H-5, the overall surface quality was significantly improved in each case. It is assumed that the larger graphene nanoplatelets (xGnP M-5 and xGnP H-5) could help efficient cutting action at the cutting angles between 90–180° by reducing friction at the tool-workpiece interface, and by keeping the tool edge sharp through lubrication effect. As a result, the uncut fiber could be decreased.

### 4.3. Inner Surface

**Figure 13:**
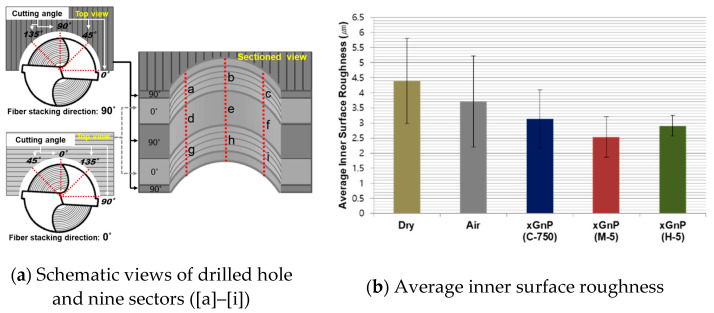
Schematic views of drilled hole and nine sectors and average inner surface roughness.

In order to investigate the MD-CFRP hole quality in further detail, the drilled holes were cut in half using a diamond wheel cutter, and sectioned parts of the workpieces were analyzed. To analyze the drilled hole surface, schematic views of the sectioned hole are shown in Figure 13a. As mentioned above, MD-CFRP with two carbon fiber stacking directions (0°, 90°) was used in this study. Based on the cutting mechanism of CFRP composites, extensive bending-induced defects would be expected to occur at the fiber cutting angle of 135°. On the other hand, with the fiber cutting angles of 45° and 90°, the carbon fiber cutting is done by the shear stress that occurs as the force is applied to the side of fiber. In the case of the fiber cutting angle of 0°, the cutting is caused by the force on the fiber being lifted by the tool [40]. Therefore, in cases with the fiber cutting angles of 45° and 90°, the carbon fiber would be adequately cut compared to that with the fiber cutting angle of 135°. Considering the general cutting mechanism of CFRP, inner surface defects of the drilled hole with a fiber stacking direction of 90° might be found on the left side, where the fiber cutting angle is 135°. By the same reasoning, in the carbon fiber stacking direction of 0°, defects could occur on the right side containing a cutting angle of 135°. In Figure 13a, nine sectors ([a]–[i]) in which the inner surface quality was investigated are shown.

To quantitatively analyze the inner surface quality, the arithmetical mean height (Ra) was measured by obtaining 3D profile data of the 90th drilled hole for each case using the 3D laser microscope (VK-X series, Keyence, Osaka, Japan) and processing software (VK Analyzer, Osaka, Japan). The device can measure fine shapes more accurately because the laser spot diameter (0.4 μm) was much smaller than a tip radius (2 μm) of a typical contact-type stylus [41]. The measurement parameter of the software was set with a cut-off length of 2.5 mm and a measurement length of 0.6 mm for nine sectors, according to the ISO 4287 standard. The measured values were averaged, and their results are shown in Figure 13b. Overall, the deviation was large because the fiber cutting angle is different in each sector. In particular, the fiber cutting angle at the sectors of [c], [d], and [i] was 135°, and the drilled surfaces were much rougher due to the bending mechanism as explained in the cutting mechanism of CFRP given in the Section 4.2.

The average surface roughness value obtained in the dry case was substantially higher than those in the other cases. On the other hand, the values decreased more when using nano-solid lubrication with graphene nanoplatelets than they did in the pure air case. Specifically, the smallest value was confirmed in the xGnP M-5 case.

**Figure 14:**
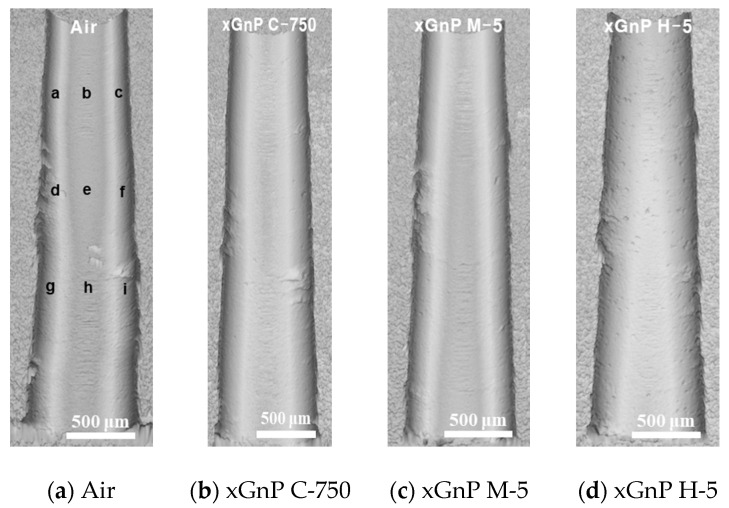
3D profiles of the 90th drilled hole and the nine sectors ([a]–[i]).

Figure 14 shows the 3D profiles that were used to analyze the inner hole surface roughness values for four experimental cases. In the case of pure air, there are some defects, such as fiber pull-out at sectors [d] and [i]. The defects at these sectors could be explained by the cutting mechanism of CFRP composite, as previously explained in Figure 13a. The same defects were found at sectors [c], [d], and [i] in the xGnP C-750 case. Meanwhile, the inner hole surface of the xGnP M-5 case was much smoother than those of the other experimental cases. In the case of xGnP H-5, defects were reduced compared to the air case, but the surface appeared to be more uneven than the xGnP M-5 case.

**Figure 15:**
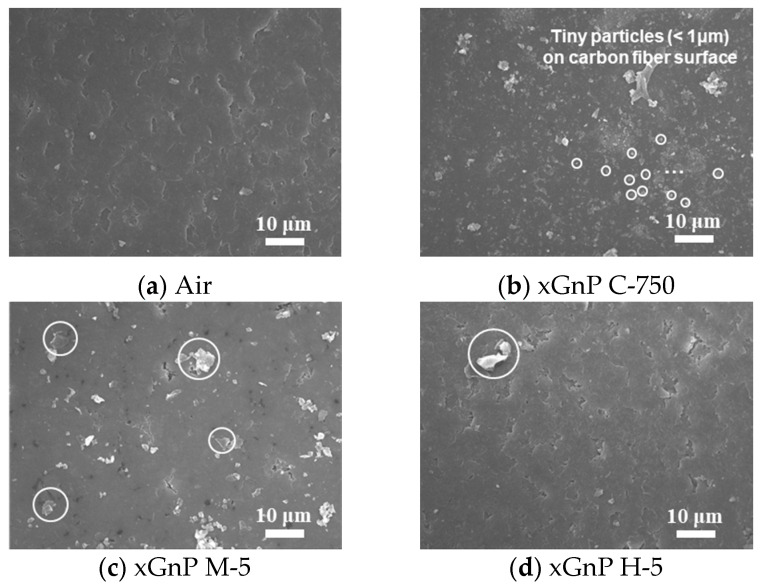
FE-SEM images of carbon fiber cutting angle of 90° (× 1400).

**Figure 16:**
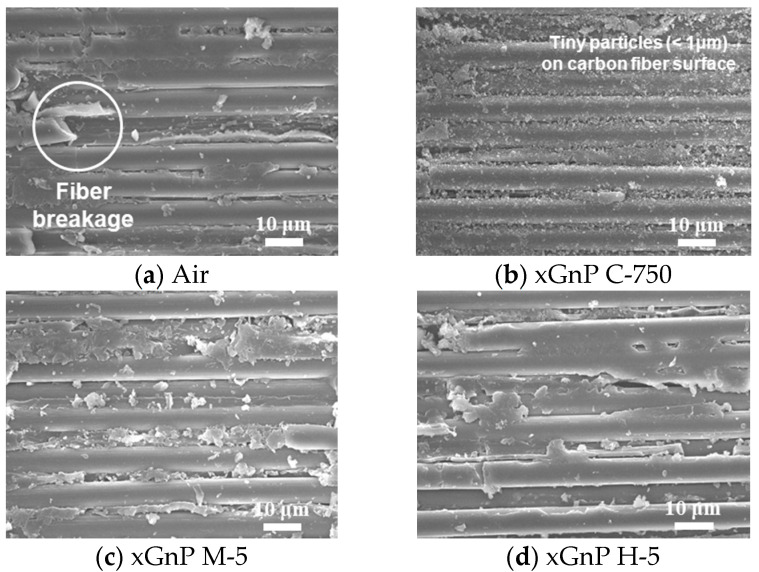
FE-SEM images of carbon fiber cutting angle of 0° (× 1400).

For more detailed analysis, FE-SEM images of the 90th hole are shown in Figure 15 and Figure 16. To investigate the carbon fiber cutting angles of 0° and 90°, images were taken at sectors [e] and [h], respectively, which are shown in Figure 13a.

As shown in Figure 15, in the air case, small debris like carbonous materials was not found on the surface, but some particles were found in the xGnP cases. In Figure 15b, a lot of tiny particles (<1 μm) could be visually identified, and this is judged as xGnP C-750 (actual size: ~0.2 μm). In Figure 15c,d, thin 2D-sheet particles were found, which seem to be attributable to xGnP M-5 and xGnP H-5, respectively (actual size of both particles: 5 μm). Therefore, it was confirmed that graphene nanoplatelets penetrated appropriately in each case during the nano-solid dry lubrication.

Figure 16 shows the inner surface at the carbon fiber cutting angle of 0°. In Figure 16a, fiber breakage was observed, which consists of defects caused by force on the fiber being lifted by a tool at a fiber cutting angle of 0°. In Figure 16b, as was the case with the fiber cutting angle of 90° shown in Figure 15b, tiny particles which were presumed to be xGnP C-750 were attached on the carbon fiber. In Figure 16c,d, it was not possible to distinguish graphene nanoplatelets from carbon dust caused by fiber breakage. FE-SEM image analysis was used to study the drilled surface morphologies according to the cutting angles of 0° and 90°, and it was also confirmed that the nano-solid lubricants properly penetrated into the drilled hole.

### 4.4. Tool Wear

**Figure 17:**
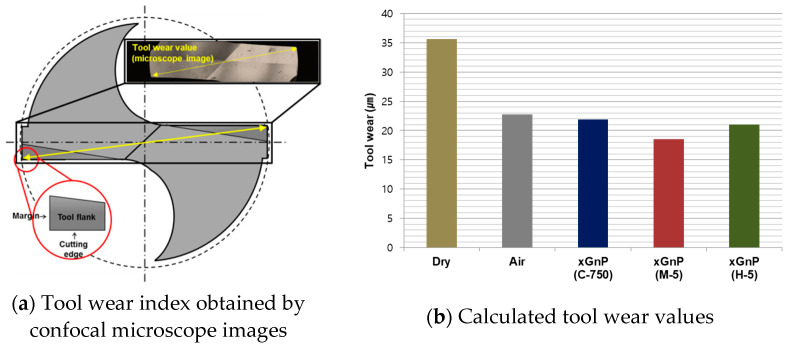
Tool wear index obtained by confocal microscope images and tool wear values.

In order to evaluate the machining performance in micro-drilling of MD-CFRP using the nano-solid dry lubrication, tool wear values were obtained and analyzed using a confocal microscope (VK-X series, Keyence) after drilling 92 holes for each experimental case. In Figure 17a, a tool wear index was marked with yellow lines for quantitative comparison. Tool wear values were obtained by calculating the difference between the diameter of a new tool and the worn drill diameter. The values indicate the occurrence of tool wear at the cutting edge and the margin of the drill bit. Therefore, the tool wear for each experimental case could be explained as the magnitude of friction between the carbon fiber and the micro-drill.

The calculated tool wear values in all experimental cases are given in Figure 17b. In the dry case, the value was even higher than those of the other cases. The tool wear value in the pure air case was also smaller than that in the dry case. The tool wear is the smallest in the case of xGnP M-5 among all cases tested. The nano-solid dry lubrication with xGnP H-5 could also be slightly effective for improving the tool wear of a micro-drill compared to the pure air case. As expected from the above results, xGnP M-5 nanoparticles, which are larger than xGnP C-750 particles, were more advantageous for reducing the tool wear. The enhanced lubrication using air spray with nanoparticles of xGnP nano-solid lubrication could lead to reduced tool wear.

**Figure 18:**
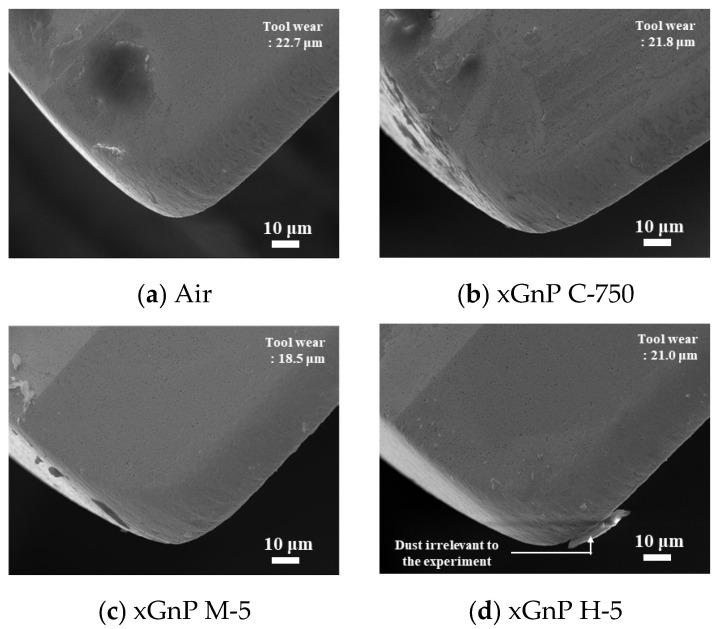
FE-SEM images of tool flank after drilling 92 holes (× 1000).

FE-SEM images of the tool flank position of Figure 17a are shown in Figure 18. All cases involved spraying air condition, in common. In the cases of pure air and xGnP C-750, adhered substance was found at the flank face of the tool. In addition, abrasive wear marks were discovered around the margin in the xGnP C-750 case. Meanwhile, in the cases of xGnP M-5 and xGnP H-5, the state of the flank face appeared relatively clean. As previously stated, larger graphene nanoplatelets could effectively reduce the friction between the CFRP composite and the tool.

### 4.5. Discussions

In order to comprehensively investigate the machining performance of micro-drilling of MD-CFRP using nano-solid dry lubrication, delamination, uncut fiber, inner surface quality, and tool wear were analyzed. Overall, nano-solid dry lubrication using xGnP C-750 improved machinability compared to the pure air case, but it was not as effective as xGnP M-5 and xGnP H-5. In other words, the larger graphene nanoplatelets (xGnP M-5 and xGnP H-5) were more advantageous than smaller particles (xGnP C-750). This could be explained in relation to how much direct contact can be effectively reduced between the tool grain and MD-CFRP workpiece. As confirmed in Section 2, it has been shown that xGnPs with a two-dimensional sheet structure lubricate while reducing the direct contact between two counterparts by creating graphene-based tribofilm during the sliding action.

**Figure 19:**
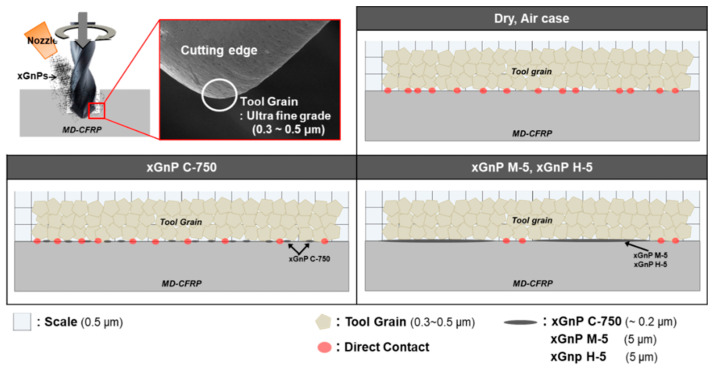
Lubrication mechanism of micro-drilling of MD-CFRP using nano-solid dry lubrication according to the average particle size (APS) of graphene nanoplatelets.

Meanwhile, the applied force was 50 N in the ball on plate test in Section 2, and all carbonous particles seemed to be smashed into even finer ones. Therefore, the measured coefficients of friction could be similar regardless of geometrical characteristics of various xGnPs, as can be seen in Figure 4a. However, in the micro-drilling experiments, the measured thrust forces were in the range between 5 N and 6 N, which is much smaller than 50 N. Therefore, the xGnP nano-scale particles might maintain their two-dimensional shapes and these geometrical characteristics could affect lubrication behavior. Thus, the lubrication mechanisms at the interface between the tool and the workpiece during the micro-drilling process were schematized in consideration of the actual sizes of the three xGnPs and the tool grain (ultrafine grade: 0.3–0.5 μm), and these schematics are shown in Figure 19. The points of direct contact between the tool grain and the MD-CFRP are marked in red.

In the cases of dry and pure air, the tool grain and MD-CFRP might contact directly without lubrication action, resulting in a smaller improvement of the machinability. The xGnP M-5 and xGnP H-5 could effectively decrease direct contact due to their larger particle size (5 μm) than that of tool grain (0.3–0.5 μm). However, in the experimental results, the difference of machining performance in the two xGnPs (xGnP M-5 and xGnP H-5) with different thicknesses was not clear. The xGnP C-750 with a smaller size (~0.2 μm) than tool grain would not have reduced direct contact as effectively as the larger xGnPs (xGnP M-5 and xGnP H-5). Therefore, in the micro-drilling of MD-CFRP, it was analyzed that the larger graphene nanoplatelets could contribute to more efficient lubrication at the moment of machining.

## 5. Conclusions

In this paper, the characteristics of micro-drilling of MD-CFRP were evaluated while considering the tribological behavior of graphene nanoplatelets as nano-solid lubricants. First, to confirm the tribological behavior of the nano-solid lubricants against CFRP composite, the ball on plate test was conducted to investigate the coefficient of friction, wear width, and wear depth. In addition to three xGnPs (xGnP C-750, xGnP M-5, and xGnP H-5), MWCNTs and hBN were also selected for comparison in this tribological test. The test results showed that the three xGnPs and the MWCNTs, which consist of 2D graphene sheets could help improve the lubrication behavior by reducing the direct contact between the two counterparts (the test ball and the specimen) due to the creation of graphene-based tribofilm. On the other hand, hBN, which consists of ceramic materials, could not lessen friction as it prevented the creation of carbon-based tribofilm.

Through the micro-drilling of MD-CFRP with the nano-solid dry lubrication, delamination, uncut fiber, inner surface quality, and tool wear were comprehensively investigated, and the lubrication mechanism in each experimental case was analyzed by reflecting the actual sizes of the nano-solid lubricants (xGnP nano-scale particles) and the tool grain according to scale. Among the three xGnPs, the larger xGnPs (xGnP M-5 and xGnP H-5) decreased surface defects and tool wear significantly, and the reduction rates were the following when compared to pure air case: Delamination—5.3%; uncut fiber—63.0%; inner surface roughness—31.7%; tool wear—18.5%. Meanwhile, the smaller one (xGnP C-750) was less effective, and the reduction rates were the following compared to pure air case: delamination—1.8%; uncut fiber—34.6%; inner surface roughness—15.5%; tool wear—3.7%.

Altogether, to guarantee lubrication effects, it was concluded that the average particle size of the graphene nanoplatelets should be sufficiently larger to reduce the points of direct contact between the tool and the CFRP.

## Figures and Tables

**Table 1:** Properties and shapes of xGnPs, MWCNTs, and hBN [19,20,21].

xGnP C-750	xGnP M-5	xGnP H-5	MWCNTs	hBN
APS: ~0.2 μm	APS: 5 μm	APS: 5 μm	Outer diameter: 10~30 nm	APS: 70 nm
Thickness: ~1 nm	Thickness: ~8 nm	Thickness: ~15 nm	Inner diameter: 5~10 nm	SSA: 19 m^2^/g
SSA: 750 m^2^/g	SSA: 120~150 m^2^/g	SSA: 50~80 m^2^/g	Length: 10~30 μm	
			SSA: >200 m^2^/g	
Shape: 2D-Sheet	Shape: 2D-Sheet	Shape: 2D-Sheet	Shape: Tube-shape	Shape: Lamella structure
Element: Carbon I	Element: Carbon I	Element: Carbon I	Element: Carbon I	Element: Boron (B), Nitrogen (N)

**Table 2:** Information on carbon fiber and prepreg [26].

Properties of Carbon Fiber and Prepreg	Information
Thickness of each ply	310 μm
Standard of carbon fiber	T700
Tensile strength	4900 Mpa
Tensile modulus	230 Gpa
Strain	2.1%
Density	1.80 g/cm^3^
Filament diameter	7 μm
Fiber area weight (FAW)	150 g/m^2^
Fiber volume	64%

## Data Availability

The data presented in this study are available on request from the corresponding author.

## Abbreviations

MD	Multi-directional
UD	Uni-directional
CFRP	Carbon fiber reinforced plastic
xGnPs	Graphene nanoplatelets
MWFs	Metal working fluids
MQL	Minimum quantity lubrication
MWCNTs	Multiwall carbon nanotubes
COF	Coefficient of friction
hBN	Hexagonal boron nitride
FE-SEM	Field emission scanning electron microscope
APS	Average particle size
SSA	Specific surface area
COF	Coefficient of friction
FAW	Fiber area weight
I_D_	Intensity of D band
I_G_	Intensity of G band
I_2D_	Intensity of 2D band
I_G_	Intensity of G band
F_d_	Delamination factor
D_nom_	Nominal diameter
D_max_	Maximum extension of delamination
A_f_	Uncut fiber area
A_nom_	Nominal drilled-hole area
A_ex_	Extracted area through image processing
R_a_	Arithmetical mean height
